# Highlights on the Role of *KRAS* Mutations in Reshaping the Microenvironment of Pancreatic Adenocarcinoma

**DOI:** 10.3390/ijms221910219

**Published:** 2021-09-23

**Authors:** Shirin Hafezi, Maha Saber-Ayad, Wael M. Abdel-Rahman

**Affiliations:** 1 Sharjah Institute for Medical Research, University of Sharjah, Sharjah 27272, United Arab Emirates; shaghani@sharjah.ac.ae; 2 Department of Clinical Sciences, College of Medicine, University of Sharjah, Sharjah 27272, United Arab Emirates; 3 Department of Medical Laboratory Sciences, College of Health Sciences, University of Sharjah, Sharjah 27272, United Arab Emirates

**Keywords:** RAS, adenocarcinoma, pancreas, tumor microenvironment, stellate cells, cancer-associated fibroblast, carcinogenesis, immunotherapy, tyrosine kinase inhibitors

## Abstract

The most frequent mutated oncogene family in the history of human cancer is the RAS gene family, including *NRAS*, *HRAS*, and, most importantly, *KRAS*. A hallmark of pancreatic cancer, recalcitrant cancer with a very low survival rate, is the prevalence of oncogenic mutations in the *KRAS* gene. Due to this fact, studying the function of *KRAS* and the impact of its mutations on the tumor microenvironment (TME) is a priority for understanding pancreatic cancer progression and designing novel therapeutic strategies for the treatment of the dismal disease. Despite some recent enlightening studies, there is still a wide gap in our knowledge regarding the impact of *KRAS* mutations on different components of the pancreatic TME. In this review, we will present an updated summary of mutant *KRAS* role in the initiation, progression, and modulation of the TME of pancreatic ductal adenocarcinoma (PDAC). This review will highlight the intriguing link between diabetes mellitus and PDAC, as well as vitamin D as an adjuvant effective therapy via TME modulation of PDAC. We will also discuss different ongoing clinical trials that use *KRAS* oncogene signaling network as therapeutic targets.

## 1. Introduction

Mutations in the *RAS* gene family are common in many cancer types. The point mutations in the Kirsten rat sarcoma viral oncogene homolog (*KRAS*) gene typically affect the hotspots at codons 12 and 13 [1,2] However, at lower frequencies, *KRAS* mutations can also occur in codons 18, 61, 117, and 146. RAS GTPase is a small guanosine triphosphatase (GTPase) that acts as a molecular switch and interacts with more than 20 effector proteins through localization to the inner surface of the cell membrane [1,2]. The point mutation in *KRAS* can impair the intrinsic GTPase activity of KRAS protein, preventing its conversion from an active form “guanosine triphosphate” (GTP) to its inactive form “guanosine diphosphate” (GDP). Consequently, KRAS remains permanently bound to GTP resulting in activation of downstream signaling pathways [1,2].

*KRAS* mutations are predominant in most cancers, such as pancreatic ductal adenocarcinoma (PDAC) (86%), colorectal cancer (CRC) (85%), and lung cancer (30%) [3]. This is followed by *NRAS* (12%) mutations, which are predominant in cutaneous melanoma and acute myelogenous leukemia. However, *HRAS* mutations that are found in bladder and head and neck squamous cell carcinomas are infrequently seen in other types of cancers [4]. According to the COSMIC v94 database, 99% of *KRAS* mutations are missense mutations, mainly with a gain of function.

In this review, we will first discuss the pathobiology of PDAC. Then, the significance of *KRAS* mutations in PDAC will be discussed. In addition, we will show how modulation of the immune response and promotion of angiogenesis by oncogenic *KRAS* can alter the tumor microenvironment (TME). We will finally highlight the link between diabetes and PDAC, as well as the importance of vitamin D for effective targeted therapies.

## 2. Pathobiology of Pancreatic Ductal Adenocarcinoma

Tumors of the exocrine pancreas are, by far, the most common type of pancreatic cancers, of which PDAC is the most common type (90%). PDAC is an epithelial tumor, and its formation requires a stepwise progression over many years. In other words, it requires the transition of a normal pancreatic duct to a pre-invasive precursor lesion, a frank malignant, invasive cancer, then a metastatic tumor. Histologically, there are three morphological noninvasive precursor lesions of PDAC, including pancreatic intraepithelial neoplasms (PanIN), intraductal papillary mucinous neoplasms (IPMN), and mucinous cystic neoplasms (MCN), of which PanIN is the most studied one. PanIN may advance to cancer that exhibit invasion, metastasis, and therapeutic resistance through a dense stromal microenvironment (desmoplastic) establishment in addition to the development of genetic variability [5]. The PDAC TME comprises a myriad of cells in addition to hyaluronic acid, cytokines, chemokines, and a variety of collagens. The cellular component includes macrophages, dendritic cells, T cells, and B cells [6]. Local immunity is always suppressed, resulting in an ideal milieu for tumor initiation, progression, as well as distant metastasis. The cold tumor with dominant CD4+ regulatory T-cells usually evades the immune system and dense desmoplastic TME hinders the access of therapeutic agents [7].

Several gene alterations have been identified during tumor progression and interaction with the TME. The whole-exome sequencing analysis of PDAC revealed around 60 genetic alterations, most of which are point mutations [8]. According to several studies, *KRAS* is the most frequently mutated oncogene in PDAC (from 70% to 95%). In addition to *KRAS*, mutations were identified in other well-known genes, e.g., *CDKN2A* (encoding p16), *TP53*, *ARID1A*, *SMAD4*, as well as in novel genes, e.g., *ATM* (one of the key genes of DNA repair), *EPC1* and *ARID2* (involved in chromatin modification), and *KDM6A* and *PREX2* (involved in carcinogenesis) [9].

*KRAS* mutations in exon 3 have a remarkably favorable prognosis. Coexistent *KRAS* mutations were detected in the same pancreatic neoplastic mass more frequently than in other tumors. *KRAS* mutations coexistent with *TP53* alterations and/or loss of SMAD4 protein herald a worse PDAC prognosis [10]. The sensitivity of endoscopic ultrasound-guided fine needle aspiration (EUS-FNA) in the diagnosis of pancreatic malignant lesions can be improved by implementing the evaluation of the *TP53* gene [11].

*TP53* alterations have been detected in 50–75% of PDACs. The disease outcome is worsened with loss of normal p53 protein, mainly if combined with *KRAS* mutations and loss of expression of SMAD4. The coexisting mutations lead to one of the aberrant signaling nodes in PDAD that shows an enhanced activity of hepatocyte growth factor receptor (HGFR) and its respective tyrosine kinase, epidermal growth factor receptor (EGFR), and an increased expression of neuropilin 1, CD44, and β1 integrin. Such activity is augmented by heterodimerization of HGFR and EGFR [12]. Approximately 50% of pancreatic cancers harbor inactivated *SMAD4* due to intragenic mutations or homozygous deletion, which occur late in PDAC. The loss of SMAD4 protein is associated with an increased risk of metastases and a worse prognosis [10,13]. In PDAC, *SMAD4* mutations result in suppression of TGF-β signal transduction and may lead to altered sensitivity to gemcitabine [11,14]. Similarly, approximately 95% of sporadic pancreatic carcinomas have inactivated *CDKN2A* as a result of intragenic mutation [15,16]. *CDKN2A* is linked to familial pancreatic cancer. Suppressed p16 expression is associated with larger tumors and with a poorer prognosis [11,17]. It is noteworthy that CDK4 inhibitors have shown promising results for the treatment of *CDKN2A*-deficient tumors in preclinical PDAC models [18]. *BRCA1/2* mutations have been identified in 5 to 10% of PDAC. Such mutations may lead to either sporadic or familial disease [8,19].

**Figure 1:**
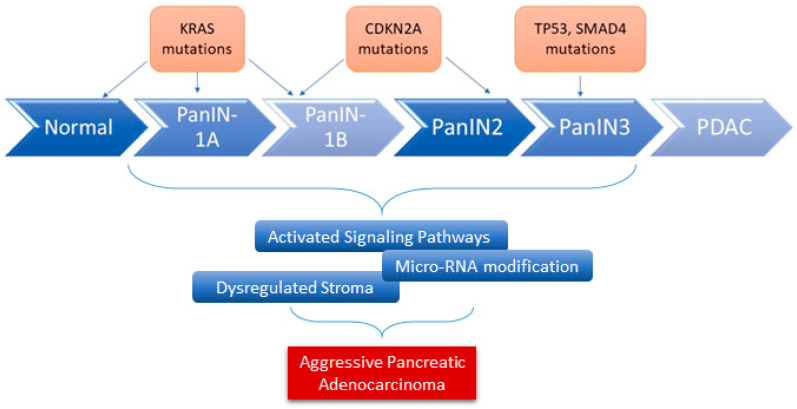
The role of genes and microRNAs in the progression of PDAC. Alterations in various key genes contribute to the progression of PDAC (overexpression, loss of function mutation, and inactivation). Many other factors have been shown to serve as fuel for the development of aggressive PDAC, including microRNAs dysregulation.

Infrequent genetic alterations and events in PDAC include microsatellite instability (MSI), also known as defective DNA mismatch repair (dMMR), BRAFV600E mutations, and MGMT promoter hypermethylation [11]. In addition to these genetic alterations, other factors serve as fuel for aggressive pancreatic cancer development. This includes dysregulated stromal-associated factors, signaling pathways, and microRNAs (Figure 1), [20].

Subgroups of PDAC were defined according to the presence of mutations/genomic alterations/events. Intriguingly, the locally rearranged subgroup is characterized by >50 events limited to one to three chromosomes. These events are typically oncogene amplifications that target existing therapeutics or genomic catastrophes such as chromothripsis [21].

## 3. *KRAS* Signaling Pathways in PDAC

Approximately 86% of somatic alterations in PDAC target *KRAS*. G12D and G12V variants account for approximately 80% of *KRAS* mutations and hence the initiation of most PDAC cases [22]. G12 mutation is followed by that of G13 (9%) and Q61 (1%) in PDAC [23]. Mutations of the *KRAS* exon 2 codons G12 and G13 exist in almost all PDAC cases (in more than 95% of PDAC cases). Other mutations, such as Q61 (<1%) in *KRAS* exon 3 and K117 and A146 (<1%) in exon 4, seem to be additional hotspots associated with constitutively activated *KRAS* in pancreatic cancer [24].

**Figure 2:**
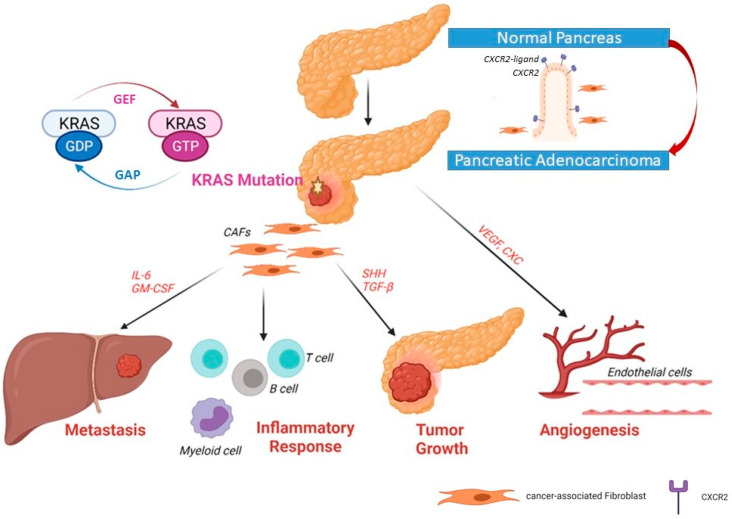
Upregulated expression of CXCR2 and its ligand in pancreatic ductal adenocarcinoma. In primary pancreatic tumors, the upregulation of CXCR2 expression induces the secretory function in cancer-associated fibroblasts (CAFs) Recruitment of fibroblasts to become CAFs can help tumor cells to grow, induce angiogenesis and invade the portal vein and metastasize to the liver.

In normal cells, the active state KRAS is bound to GTP, while it is bound to GDP in the inactive state. RAS proteins keep switching “on” and “off” through conformational changes through binding of GTP and GDP. GEF (guanine nucleotide exchange factor) promotes dissociation of GDP and acts as a positive regulator; GAP (GTPase-activating protein) promotes hydrolysis of GTP and acts as a negative regulator helping to keep most of KRAS in an inactive GDP-bound state (Figure 2) [25]. Most RAS mutations involve GAP-mediated inactivation of RAS. For example, substitutions in residues G12 prevent van der Waals bond formation between RAS and the GAP, leading to perturbation of Q61 (or the catalytic glutamine) orientation in RAS. This results in the pronounced attenuation of GTP hydrolysis, with enduring activation of RAS-driven downstream pathways [26]. Activated *KRAS* induces a myriad of downstream signaling pathways and effector proteins, such as mitogen-activated protein kinase (MAPK)–MAPK kinase (MEK), phosphoinositide 3-kinase (PI3K)–AKT–the mechanistic target of rapamycin (mTOR), rapidly accelerated fibrosarcoma (RAF)–MEK–extracellular signal-regulated kinase (ERK), and Nuclear factor-κB (NF-κB) pathway (among other nuclear transcription factors). These factors can enhance the survival, proliferation, transformation, and invasion of cancer cells [27]. Additionally, mutant KRAS results in the autonomous release of type I cytokine complexes by cancer cells. Subsequently, a cascade of events follows that leads to metabolic reprogramming (see Section 5) [28]. The signaling pathways of KRAS are discussed comprehensively in previous review articles [27,29,30,31]. The aforementioned studies point to the potential role of *KRAS* mutations in modulating the immune status of the TME.

## 4. Mutated *KRAS* and the Tumor Microenvironment

The modulation of the immune response through several cytokines, as well as the promotion of angiogenesis by oncogenic *KRAS,* can alter the TME [27]. *KRAS* mutations are likely to coexist with mutations of other genes in PDAC, as previously described. The summative effect on the TME shapes the immune status of the tumor surrounding, a crucial factor that determines the capacity of the tumor to metastasize and to respond to therapeutic agents [21]. As an example, a worse PDAC prognosis is expected when *KRAS* mutations coexist with *TP53* alterations and/or loss of SMAD4 protein. In addition, the combination of *KRAS* mutations and loss of SMAD4 enhances the activity of HGFR and EGFR, together with an increased neuropilin 1, CD44, and β1 integrin expression [21,32,33].

Yu and coworkers (2015) showed that RAS signaling regulates pathological inflammation in severe acute pancreatitis. Their study indicated that RAS signaling controls CXC chemokine formation, indirectly affecting neutrophil recruitment and tissue injury in the inflamed pancreatic tissue. Inhibition of RAS signaling resulted in the decreased taurocholate-induced pancreatic activity of myeloperoxidase, which indicates the suppression of neutrophil recruitment [34]. KRAS was involved in CXC chemokine formation and the induction of VEGF, which plays a critical role in pancreatic angiogenesis. Furthermore, RAS was shown to upregulate COX2, which, in turn, promotes tumor formation via MEK/c-Jun pathway and human umbilical vein endothelial cells (HUVEC) invasion [34,35].

### 4.1. Inflammatory Chemokines, Cytokines, and Interleukin 6

Several chemokines have been implicated in inflammation-induced tumorigenesis. The induction of several inflammatory cytokines and chemokines responsible for tumorigenesis and invasiveness has been tightly linked to oncogenic *KRAS* [36]. C-X-C motif chemokine receptor 2 (CXCR2) controls a major inflammatory signaling network in pancreatic cancers with *KRAS* mutation [37]. Previously, it has been reported that CXCR2, which is a receptor for a group of C-X-C cytokines, can enhance granulocyte recruitment to the site of inflammation, as well as enabling angiogenesis through recruitment of myeloid-derived suppressor cells (MDSCs) and immunosuppressive neutrophils [38].

*KRAS* mutations influence the stellate cells/pluripotent stem cells of the pancreas (activated stellate cells are referred to as cancer-associated fibroblasts or CAF). CAFs are one of the key players that promote tumor proliferation, migration, invasion, and metastasis. Furthermore, CAFs modulate the tumor immune microenvironment and modify its response to treatment. Thus, CAFs delineate the acquisition and maintenance of numerous cancer hallmarks [39,40]. Recently, it was demonstrated that CXCR2 can induce secretory function in CAFs via NF-κB activation. CAFs make up a united heterogeneous population of cells that can alter the microenvironment of the tumor and thus alter the neoplastic cell’s fate [37]. CAFs play a major role in the formation of the desmoplastic stroma as well [41].

CAFs can secrete many extracellular matrix proteins, such as collagen, fibronectin, and laminins, into the TME following their activation [42]. During carcinogenesis, CAFs can produce inflammatory mediators such as CXCL8 and interleukin-6 (IL-6), both of which are associated with inflammation, tumor growth, and angiogenesis [43,44]. Thus, KRAS/CXCR2 signaling plays a major role in regulating the CAFs of PDAC. Another chemokine called chemokine C-C motif ligand 5 (CCL5) is expressed by many cell types such as immune cells, epithelial cells, fibroblasts, and more importantly, by tumor cells [45]. A study by Singh et al. (2018), showed that the capacity of pancreatic cancer cells to migrate and invade other organs increases via activation of CCR5 by CCL5 that triggers a cascade of signaling pathways [46]. In another recent study, the authors discovered that CCL5 can mediate the influx of CD4+ T cells into the TME following treatment with CD40 antibody [47]. CD4+ T cells were shown to have a negative role in tumor immunity and response to immunotherapy [48]. These studies suggest that therapeutic targeting of inflammatory chemokines might result in improved outcomes in *KRAS* mutant cancers.

Interestingly, two key drivers of PDAC tumors, oncogenic *KRAS* and hypoxia, have been shown to induce IL-6 [49]. IL6 secretion has been identified to be the most characterized cytokine in PDAC, which is strongly associated with tumor survival. Its secretion can be induced both by myeloid cells from the surrounding stroma and tumor cells [50,51]. Moreover, strong phosphorylation of signal transducer and activator of transcription 3 (STAT3) induced by IL-6 resulted in PanIN-PDAC progression in K-RasG12D mice [50]. IL-6 has been shown to have a role in tumor formation. According to Zhang et al. (2013), genetic deletion of IL-6 resulted in a reduction in PanIN formation, when *K-Ras* mutation was initiated embryonically in an inducible *K-Ras*-driven mouse model. The study also showed a significant decrease in the percentage of intra-tumoral cancer-promoting macrophages and MDSCs following the deletion of IL-6 in this *K-Ras*-driven PDAC mouse model [51]. *KRAS* mutations as therapeutic targets in CAFs will be discussed later in this review [52].

### 4.2. Mutated KRAS Effect on the Surrounding Stromal Cells

Tape and coworkers (2016) showed that *KRAS*G12D communicates with stromal cells and renders tumor cells insensitivity to many important factors. These authors demonstrated that the secretion of growth factor sonic hedgehog (SHH), granulocyte colony-stimulating factor (GCSF), and granulocyte-macrophage colony-stimulating factor (GM-CSF) cytokines can be increased by active oncogenic *KRAS*G12D [52]. Hedgehog (Hh) signaling which is known to play a crucial role in embryonic development, stem cell regulation, and adult tissue homeostasis, is highly activated in PDAC [53]. SHH is a ligand of the hedgehog signaling pathway. An increase in SHH secretion via the NF-κB pathway and *KRAS* leads to the disruption of primary cilium of PDAC cells and upregulation of many extracellular matrix components, such as collagen, MMPs, and fibrillin-1. Pancreatic stellate cells (PSCs) cross talk with tumor cells to enhance local tumor growth and promote distant metastasis. It is noteworthy that PSCs represent a major origin of fibrosis in the TME [54]. SHH can alter the PSC intercellular signaling potential through upregulation of two specific growth factors: insulin-like growth factor 1 (IGF1) and growth arrest-specific gene 6 (GAS6). Via SHH, *KRAS*G12D PDAC cells can send signals to PSC and, at the same time, remain insensitive to autocrine SHH. This results in further production of IGF1 and GAS6. Consequently, these two growth factors are capable of activating the receptor tyrosine kinases (RTKs), IGF1R, and AXL [52]. This will eventually lead to increased proliferation, and resistance to apoptosis.

The overexpression of a high molecular weight glycoprotein called Mucin was shown to be associated with progression in many tumors, including PDAC [55]. Mucin can be divided into two major groups: (1) a membrane-bound mucin called MUC4 that is implicated in cell–cell and cell–extracellular matrix interactions and (2) secreted mucins that participate in epithelial protection [55]. Interestingly, aberrant activated *KRAS* in PDAC can activate and cause upregulation of this membrane-bound mucin MUC4 both at the transcriptional and post-transcriptional level via p42/44 MAPK and NF-κB pathways and RalB pathway, respectively. It has been reported that there is a direct interaction between the promoter of MUC4 with c-Fos (activated by p42/44 MAPK pathway), c-Jun, and p65 NF-κB subunit, suggesting a link between the gradual increase in both *KRAS* signaling (MAPK and NF-κB) and MUC4 expression in pancreatic carcinogenesis [56]. Moreover, silencing of RalB GTPase in PanIN lesions leads to the inhibition of MUC4 protein overexpression with no effect on its mRNA level, whereas RalA silencing has no effect on its protein expression [56].

### 4.3. Mutated KRAS Interaction with the Immune Cells

As previously mentioned, PDAC cells harboring mutant *KRAS* can secrete chemokines (e.g., GM-CSF and IL-6). These chemokines stimulate various immune cells, including T-cells and B-cells, MDSCs, and macrophages, resulting in an inflammatory TME. In addition, oncogenic KRAS stimulates the release of angiogenic factors (e.g., VEGF) [27,29]. These factors can determine the TME immune status, the possibility of tumor metastasis, and the response to treatment.

Immune evasion is a major obstacle to cancer treatment. It was found that PDAC cells lack the expression of cytokeratin 19 (CK19) and display a reduced expression of MHC-I at the cell surface. Additionally, autophagy-related genes were found to be enriched in MHC-I negative PDAC cells that reside in liver metastasis [57]. In PDAC, surface MHC-I is decreased via the NBR1-mediated autophagy–lysosomal pathway. Recently, it was shown that the surface levels of MHC-I can be restored through inhibition of autophagy [58]. This inhibition in syngeneic host mice also leads to the enhancement of antitumor T cell responses and consequently reduction in tumor growth.

It has been reported that adipose tissues, in which tumors have a predilection to grow, can convert tumor-suppressive NK cells to tumor-promoting cells through decreasing NK-mediated cytotoxicity and IFN-γ secretion and increasing IL-6 secretion, aiding tumor growth and expansion. According to Kaur et al. (2018), NK cells and monocytes are recruited to the peri-pancreatic and pancreatic adipose tissue from the circulation, where they lose the secretion of IFN-γ, while increasing the secretion of IL-6, thus perpetuating the tumor inflammatory milieu [59].

## 5. *KRAS* Mutation and Metabolic Reprogramming

Proliferating cancer cells increase the glycolysis process through the upregulation of many glycolytic proteins, because they require an increased amount of energy [60]. It has been reported that *KRAS* G12D cancer cells can increase the uptake of glucose and production of lactose, which ultimately results in glycolytic flux. They do so via upregulation of glucose transporters such as glut1/Slc2a1, enzymes of the hexosamine pathway (Gfpt1), nonoxidative pentose phosphate pathway (PPP) enzymes (Rpia and Rpe), and crucial glycolytic enzymes (Hk1, Hk2) [61]. While proliferative primary tumors rely heavily on glycolysis, metastatic tumor cells have drastically different metabolic requirements [62].

Recently, it was shown that oncogenic *KRAS* can regulate hormone-sensitive lipase (HSL), which, in turn, controls and regulates the storage of lipids for metastatic pancreatic cells [63]. Considering the evident effect of oncogenic *KRAS* on the energy production and metabolic pathways in PDAC, the question is whether such an effect extends to affect the TME.

Recently, it was shown that *KRAS* mutation mediates an autocrine effect that results in upregulating a specific type I cytokine receptors, namely IL4rα and IL13rα, dimerized to IL2Rγ. This was supported by in silico evidence via “digital microdissection” of the PDAC datasets from the Cancer Genome Atlas (TCGA) and Oncomine, which showed that the expression of IL2Rγ and IL4R are not only expressed in PDAC cells but also on the surface of various immune cells, including T-cells, basophils, eosinophils, and macrophages [64,65,66,67]. It was also shown that IL4Rα mediates the effect of IL4 and IL13 arising from the host Th2 cells in the TME. As a consequence, IL4 and IL13 activate the JAK1-STAT6-MYC pathway, thus leading to metabolic reprogramming (glycolysis pathway activation and increasing the glucose utilization by cancer cells) [28,68]. IL4 stimulation using a *Pdx-Cre*–*LSL-Kras^G12D^* model induced an increase in the tricarboxylic acid (TCA/Krebs) cycle intermediates and decreased PPP intermediates, a typical consequence of MYC activation. Exploring the intricate link between the PDAC cells and the TME at the metabolic level enables a deeper understanding of the mutual paracrine effects of the PDAC and the immune cells in the TME. It is noteworthy that IL4 also plays a central role in tumor progression via M2 polarization of macrophages that create an immune-suppressive status [69]. Furthermore, KRAS promotes MYC stability through phosphorylation, thus inhibiting its proteasomal degradation [70] and augmenting the effect of TME-derived cytokines.

## 6. *KRAS* Mutation in Patients with Diabetes Mellitus

**Figure 3:**
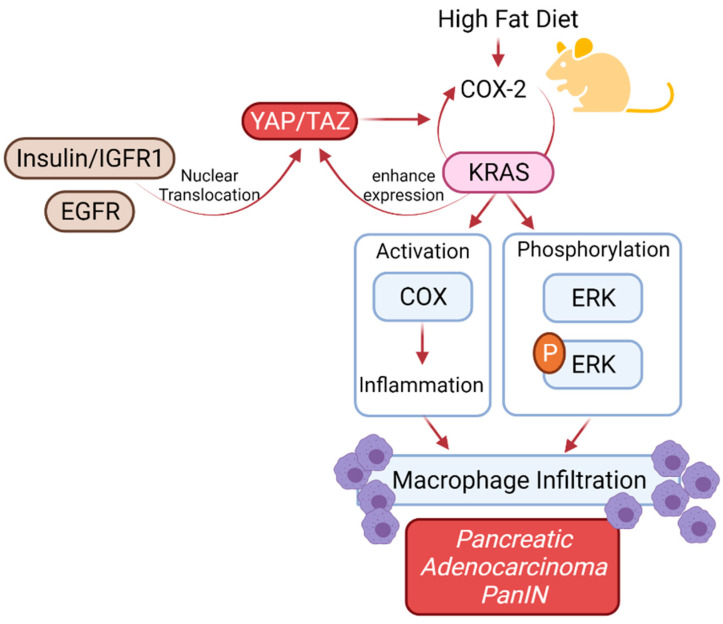
A high-fat diet (HFD) can initiate the transformation of normal pancreatic cells into PanIN lesions. This *KRAS* activation via HFD leads to downstream activation of COX2 and ERK phosphorylation, thus resulting in infiltration of macrophages into the stroma and formation of a pro-tumorigenic microenvironment.

Many factors are involved in the development of fully invasive PDAC. These include *KRAS* mutations in addition to many others, metabolic and environmental stressors, and obesity [71]. Changes in the TME, including gut microbiota, inflammation, intestinal peptides, and insulin resistance, which are associated with obesity, can enhance the activation of KRAS. A high-fat diet (HFD) can initiate the transformation of normal pancreatic cells into PanIN lesions through stimulation of oncogenic KRAS [72]. Previous studies have reported that HFD consumption helps KRAS to recruit more inflammatory mediators to the pancreas enhancing PanIN formation [72]. This KRAS activation via HFD leads to downstream activation of COX2 (positive feed-forward loop maintaining KRAS activity), phospho-ERK, and infiltration of macrophages into the stroma, which, as a result, increases inflammation in acinar cells, thus helping in PanIN formation (Figure 3) [72]. Additionally, it has been identified that YAP/TAZ transcriptional co-activators represent a major element in this amplification loop. Importantly, YAP nuclear localization is stimulated by GPCRs, EGFR, and insulin/IGF-1 receptor signaling, whereas YAP expression is enhanced by *KRAS* activation. YAP, in turn, leads to PDAC survival through BIRC5 and the evasion of immune surveillance through CXCL5 [71].

### 6.1. Therapeutic Targets in KRAS-Mutated Pancreatic Cancer

To our knowledge, there are almost no effective targeted therapies for PDAC targeting RAS signaling yet. This is because the accomplishment of RAS signaling and activation is primarily through protein–protein interactions, which are difficult to target with small molecules, since the binding pocket is not well defined [4,73]. Furthermore, immunotherapy has had minimal clinical success in pancreatic cancer; thus, it is not yet included in the clinical guidelines. The lack of efficacy of immunotherapy may be explained by the "cold" character of these tumors, being infiltrated by few lymphocytes, as well as the complexity of their TME. As a result, ongoing clinical studies are focused on combinatorial methods that target the immune system (e.g., PD-L1) and pancreatic TME molecular inductors (e.g., colony-stimulating factor receptor 1 (CSFR1), chemokine C-X-C receptor 4 (CXCR4), and others). It is anticipated that reprogramming the TME, possibly by targeting the *KRAS* mutations, may increase the treatment efficacy [74].

The earliest identified RAS-binding small molecules were able to bind to the hydrophobic pocket on the CDC25 domain of SOS. At a low micromolar concentration, these molecules have been shown to increase RAS-GDP levels and thus disrupt MAPK and PI3K signaling [73]. Shortly after, Kobe0065-family compounds were found to inhibit RAS protein–protein interactions and its downstream effectors through binding to the RAS-GTP site [75]. Subsequently, a third class of small molecules for RAS inhibitor called SML-8-73-1 was developed. This GDP analogue was shown to be able to specifically target PDAC cells with a *KRAS*G12C missense mutation via competition with GTP and GDP for active site binding. However, due to many reasons, it was not successful. Another G12C inhibitor (MRTX849, adagrasib) has shown therapeutic benefit in NSCLC and could represent a good option for PDAC treatment, awaiting the completion of the KRYSTAL-1 study. It should be emphasized that this mutation (G12C) is rarely found in PDAC compared to G12D and G12V mutations; it is rather more common in non-small-cell lung cancer. Furthermore, targeting this mutation has a potential off-target activity due to its high concentration requirement [76,77,78]. It is noteworthy that MRTX1133 is a novel potential first-in-class “G12D” inhibitor that has progressed through investigational new drug (IND)-enabling studies in colorectal and pancreatic cancer.

Another strategy to disrupt and prevent RAS function is through interference with the binding of phosphodiesterase 6 delta (PDEδ) to KRAS, thus hindering tumor development. PDEδ is responsible for the recognition of *KRAS*4B and its transition to the plasma membrane. It was demonstrated that the inhibition of the PDEδ–KRAS interaction by a small molecule called deltarasin reduced the growth of *KRAS*-dependent PDAC cell lines [79]. Furthermore, deltarasin was shown to decrease proliferation and increase apoptosis in *KRAS* mutated pancreatic tumor cells through the blockage of PDEδ–*KRAS* interaction, thus preventing their membrane localization in these cells. Table 1 summarizes the clinical trials of potential therapies targeting KRAS and its signaling pathways for the treatment of pancreatic ductal adenocarcinoma.

### 6.2. Therapeutic Targets of KRAS Mutation in CAFs and Importance of Vitamin D Therapy

PDAC cells with *KRAS* mutation can increase the secretion of myofibroblast content and the desmoplastic reaction through signaling to pancreatic CAFs via SHH secretion [80]. SHH can activate insulin-like growth factor 1 (IGF1), its receptor (IGF1R), and AXL [52]. Interestingly, the pro-tumorigenic phenotypes caused by paracrine signaling between PDAC cells and SHH-activated CAFs can be reversed via AXL pharmacological inhibition. An ongoing clinical trial is testing the addition of bemcentinib (BGB324), a first class selective oral inhibitor of AXL to nab-paclitaxel/gemcitabine/cisplatin in the treatment of PDAC [81], and another trial is using another Axl inhibitor (TP-0903) [82]. The results of those trials are crucial to evaluate targeting the TME in PDAC.

Additionally, the activated CAFs release the CXCL12 chemokine, which binds to one of its two receptors: ACKR3 and CXCR4: [83]. The inhibition of CXCR4–CXCL12 interaction can increase tumor sensitivity to anti-PD-1 ligand-1 (PD-L1) therapy and enhance T cell access to the TME [84]. BL-8040 is a small synthetic peptide that binds to CXCR4 with a very high affinity. This CXCR4 inhibitor has demonstrated a longer receptor occupancy compared to other CXCR4 inhibitors, such as Plerixafor (AMD3100) [85]. Recently, it was shown that combined PD-1 and CXCR4 inhibitors treatment on PDAC tissues can increase the tumor cell apoptosis and CD8+ T cell migration into the juxta-tumoral compartment [84].

CAFs can increase vitamin D receptor (VDR) expression and decrease the expression of lipid storage genes in PDAC. Increased stromal expression of α-smooth muscle actin (αSMA) by VDR correlates with aggressive pancreatic cancer biology [86]. Upon treatment with a synthetic form of vitamin D called calcipotriol, CAFs hindered epithelial-to-mesenchymal transition (EMT), decreased the chemoresistance, increased lipid storage gene expression, and hindered the action of myeloid-derived suppressor cells (MDSCs) [87]. On the contrary, the downregulation of VDR can trigger EMT by many factors, such as cytokines and several cellular signaling pathways, including β-catenin. The reversal of EMT, drug resistance, and metastasis has been achieved via the use of VDR agonists [88]. Recently, it was shown that calcipotriol can reduce the tumor supportive activity of CAFs [89]. In this study, in response to vitamin D, upregulation of PD-L1 in CAFs was observed. On the other hand, the expression of PDL-2 expression in CAFs was decreased. the upregulation of PD-L1 was shown to influence the T cell-mediated tumor immune surveillance [89].

### 6.3. Modulating the Immune Status of PDAC Microenvironment

A cell surface marker called programmed death-1 (PD-1) and its ligand PD-L1 have been established as targets for blockade in the immunotherapy of many solid tumor types. These cell surface markers were shown to be involved in many regulatory checkpoint pathways [92,93]. However, as single agents, their blockers have limited activity for PDAC. According to a study by Kim et al. (2020), the authors showed for the first time that, within the TME, a listeria vaccine-based ANXA2-targeting cancer immunotherapy (Lm-ANXA2) was capable of inducing tumor epitope-specific CD8+ T cell response and sensitizing the PDAC tumor to checkpoint inhibitor therapy [94].

Annexin-2 (ANXA2) is a calcium-dependent phospholipid-binding protein that presents as a hetero-tetramer with S100A10 on the cell membrane and in the cytoplasm. It plays a major role in exocytosis, endocytosis, membrane trafficking, and cellular cytoskeleton upon phosphorylation, as well as cellular growth and signaling pathways [95,96]. Previous studies demonstrated that ANXA2 plays a crucial role in the development of many cancer types including PDAC. This protein has been shown to cause cancer cell proliferation, invasion, migration, and, most importantly, angiogenesis and metastasis through facilitating extracellular matrix (ECM) degradation [95,96]. Additionally, ANXA2 was associated with chemotherapy resistance in PDAC via upregulation of the NF-κB pathway [97]. Interestingly, the anti-tumor cytokine IFNγ-expression by T cells was significantly enhanced through the combination of anti-PD-1 antibody with Lm-ANXA2 vaccine therapy [94]. This combination therapy also resulted in prolonged survival in genetically engineered KPC mice (having *KRAS* and P53 mutations) with spontaneous PDAC tumors.

The expression and function of proliferative Yes-associated protein (YAP1) have shown to be upregulated in *KRAS* mutated PDAC through the atypical protein kinase C isoform ι (PKCι), leading to the progression of PDAC, reprogramming of microenvironment, and immune invasion of PDAC [98]. PKCι has been shown to upregulate another important protein called Specificity protein 1 (Sp1). This protein is the first identified member of the Sp/XKLF (specificity protein/Krüppel-like factor) family of transcription factors shown to modulate apoptosis, differentiation, angiogenesis, and growth of many different cell types [99]. It has been reported that upregulated Sp1via PKC1 can bind to multiple sites of YAP1 promoter driving its transcription, which ultimately leads to upregulation of PDl-1 and thus the proliferation of PDAC as well as cytotoxic immune response resistance [98]. The induction of apoptosis and reversion of the immunosuppression of pancreatic cancer cells was accomplished through the combination of PKCι and Sp1 inhibitors at sub-toxic doses. The synergistic effect of this combination has been shown to sensitize PDAC to the cytotoxicity of natural killer (NK) cells. Interestingly, significant suppression of PDL1 expression in PDAC was also achieved through this combination therapy [98].

## 7. Conclusions

Mutations of *KRAS* appear to alter the immune microenvironment composition of PDAC in addition to their established role in the disease initiation and progression. The effect of mutant *KRAS* on the TME is mediated via several pathways/mechanisms, including cytokine secretion, interaction with the immune cells and CAFs, and metabolic reprogramming. The investigation of these pathways will not only improve our understanding of tumor-immune evasion but also will help developing new biomarkers and improving the outcome of immunotherapy in PDAC. A recognized mutual link between diabetes mellitus and PDAC was observed, with the notorious effect of a high-fat diet on modulating immune cell recruitment to the TME. Furthermore, Vitamin D may hinder metastasis by suppressing epithelial–mesenchymal transition through its action on CAFs. Combination treatments targeting these *KRAS*-regulated pathways that trigger the establishment of an immune-suppressive milieu might help patients respond better to currently available immunotherapies. Targeting *KRAS* mutations may give rise to potential treatment strategies for the unresolved problem of pancreatic cancer.

## Figures and Tables

**Table 1:** Clinical trials of potential therapies targeting KRAS and its signaling pathways for the treatment of pancreatic ductal adenocarcinoma.

Targets/Diseases	Drugs	Phase of Trial	Patients/ In Vivo/In Vitro	Outcomes	References
Targeting CXCR4 in PDAC	BL-8040 (CXCR4 inhibitor) plus pembrolizumab with or without 5-FU and liposomal irinotecan	Phase 2	80 Patients	Objective response rate	[90] NCT02826486 [91]
Targeting AXL	(Nab-paclitaxel, Gemcitabine, Cisplatin) with or without BGB324 (Axl inhibitor) TP-0903	Phase 1 and 2 Phase 1	74 Patients 177 Patients	Decreased tumor volume and increased cancer cell apoptosis	NCT03649321 [81] NCT02729298 [82]
Metabolism in RAS-driven Pancreatic cancer. Stage II, III, IV pancreatic cancer	Trametinib, hydroxychloroquine	Phase 1	33 participants	Ongoing Results are not yet available	NCT03825289
Targeting autophagy/Metabolism in RAS-driven Pancreatic cancer. Metastatic pancreatic adenocarcinoma, stage IV pancreatic cancer	Hydroxychloroquine, binimetinib	Phase 1	39 participants	Ongoing Results are not yet available	NCT04132505
KRAS p.G12C Mutant Advanced Solid Tumors	AMG 510 (Sotorasib) Anti PD-1/L1 Midazolam	Phase 1 and 2	733 participants	Partial responses in two of four NSCLC patients, with stable disease achieved in the remaining two	NCT03600883
Multiple clinical trials are underway to assess the benefit of vitamin D treatment in PDAC					
Multiple clinical trials are underway to assess the benefit of vitamin D treatment in PDAC	calcipotriol (a synthetic form of vitamin D) Combined Calcipotriol and gemcitabine treatment		In vivo In vivo	Reduced markers of inflammation and fibrosis in pancreatitis and human tumor strom aEnhanced the survival of the KPC (*KRAS*LSL-G12D/+; Trp53LSL-R172H/+; Pdx-1-Cre) mouse model, ultimately increasing median animal survival by 57%.	NCT03472833 NCT03300921 NCT02754726 [87]
Targeting vitamin D receptor (VDR) /PDAC	Vitamin D receptor agonist paricalcitol plus gemcitabine and nab-paclitaxel in patients with metastatic pancreatic cancer		Phase 2	112 Patients	Ongoing Results are not yet available

## Data Availability

Not applicable.

## Glossary and Abbreviations

AXL	Is a member of the TAM (Tyro3, Axl, MerTK) receptor tyrosine kinase family. It has been found to drive metastasis and cause immune suppression in different cancers including PDAC.
CAFs	Cancer-associated fibroblasts. It can promote tumorigenic features by initiating the remodeling of the extracellular matrix by secreting cytokines.
ERK	MEK–extracellular signal-regulated kinase.
FoxM1	Fork-head box M1.
GAS6	Growth arrest-specific gene 6 (GAS6). It has an important role in the stimulation of cell proliferation.
IGF-1	Insulin-like growth factor 1 (IGF1), is a hormone that plays an important role in childhood growth. Through inhibition of apoptosis, IGF-1 has been shown to promote cancer development.
INK4a–ARF	Inhibitors of CDK4 (INK4). The INK4a–ARF locus on chromosome 9 is one of the sites mutated most frequently in human cancer. Two genes comprising over-lapping reading frames encoding p16 (INK4a) and p19 (ARF) have been discovered at this locus, and remarkably, both play an important role in regulating cell growth, survival, and senescence.
KRAS	Kirsten rat sarcoma viral oncogene homolog (KRAS) gene: A gene that makes a protein that is involved in cell signaling pathways that control cell growth, cell maturation, and cell death. The natural, unchanged form of the gene is called wild-type KRAS. Mutated (changed) forms of the KRAS gene have been found in some types of cancer, including non-small-cell lung cancer, colorectal cancer, and pancreatic cancer. These changes may cause cancer cells to grow and spread in the body.
MAPK	mitogen-activated protein kinase.
MEK	MAPK kinase.
MHC	The major histocompatibility complex (MHC) is a large locus on vertebrate DNA containing a set of closely linked polymorphic genes that code for cell surface proteins essential for the adaptive immune system. PDAC cells show a reduced expression of MHC-Class1 on their cell surface.
mTOR	mechanistic target of rapamycin.
MUC4	Mucin4 is a large membrane-anchored glycoprotein that belongs to the mucin family. They play an important role in the protection of epithelial cells. Its overexpression has been seen in many types of carcinomas.
NF-κB	Nuclear factor- κB.
PI3K	phosphoinositide 3-kinase.
PPP	pentose phosphate pathway.
RAF	rapidly accelerated fibrosarcoma.
SHH	Sonic Hedgehog (SHH), is one of the hedgehog pathways that play an important role in the regulation of embryonic development. It has been found to play an important role in tumor initiation and invasiveness.
SMAD4	Mothers against decapentaplegic homolog4 (SMAD4), also called DPC4, is an Intra-cellular messenger of TGF-β and shows an anti-tumor effect by inhibiting the cell growth.
TCA	tricarboxylic acid.
VDR	Vitamin D receptor.
